# Malaria in the eco-epidemiological region of the Colombian Caribbean, 1960-2019

**DOI:** 10.17843/rpmesp.2022.394.11359

**Published:** 2022-12-05

**Authors:** Luis Acuña-Cantillo, Mario J. Olivera, Julio Cesar Padilla-Rodríguez

**Affiliations:** 1 Instituto Nacional de Salud de Colombia, Bogotá, Colombia. Instituto Nacional de Salud de Colombia Bogotá Colombia; 2 Knowledge Management, Research and Innovation in Malaria Network, Instituto Nacional de Salud de Colombia, Bogotá, Colombia. Knowledge Management, Research and Innovation in Malaria Network Instituto Nacional de Salud de Colombia Bogotá Colombia

**Keywords:** Malaria, Epidemiology, *Plasmodium vivax*, *Plasmodium falciparum*, Caribbean, Colombia

## Abstract

Malaria has a heterogeneous and variable behavior among Colombian regions. In order to establish its epidemiological behavior in the Colombian Caribbean region between 1960 and 2019, we carried out an observational, descriptive and retrospective study based on records from the Ministry of Health and other secondary sources. We defined epidemiological variables and used measures of frequency and central tendency. A total of 155,096 cases were registered. The decades with the highest number of cases were 1990-1999 (20.5%) and 1980-1989 (18.9%). The average number of cases per decade was 25,849.3. The highest parasite rates were recorded in 1970 (3.3 per 1000 population) and 1981 (3.9 per 1000 population). Plasmodium vivax was the most frequent species and most of the burden by age group was found in people under 29 years of age, between 2010-2019. Malaria showed an endemic-epidemic pattern of low and very low transmission intensity, with a decreasing trend.

## INTRODUCTION

In Colombia, malaria accounts for 54.7% of the cumulative burden of cases of vector-borne diseases^ (^ ^1^. In the last five years, there has been a change in the prevalence of the parasitic species in the country, with *Plasmodium falciparum *being the most frequent one ^2^ ^,^ ^3^.

This disease persists as a public health problem, imposing a high economic and social burden ^4^ ^,^ ^5^. Its transmission is endemic-epidemic, heterogeneous and varies from medium to low intensity in the eco-epidemiological regions throughout the national territory, under different conditions of receptivity and vulnerability.

In the Caribbean region, the disease is confined and of very low transmission^ (^ ^6^. In 2018, it contributed 3% of the national malaria burden, and most cases were imported from other regions with varying degrees of transmission intensity ^7^. Despite this, few studies have been carried out on the subject in this region, which have been specific and with limited dissemination, making it difficult to make evidence-based decisions for the definition and implementation of eradication strategies ^8^.

The intensification of environmental, economic, social, political, and cultural interactions in the last decade could change the transmission dynamics in the Caribbean region in the coming years, favoring the reemergence, continuity, and intensification of endemic-epidemic levels in the region ^9^. Therefore, the aim of this study was to identify the epidemiological behavior of malaria in the Colombian Caribbean region between 1960 and 2019.

**KEY MESSAGES**

Motivation for the study: the information available on the epidemiology of malaria in the Colombian Caribbean region is incomplete, poorly systematized and its dissemination is limited. This has led to a lack of knowledge of its magnitude and a low perception of its importance as a public health problem.

Main findings: the behavior of malaria is endemic-epidemic, with low to very low transmission, focused and with irregular outbreaks. Plasmodium vivax infections predominate.

Implications: .the results of this study contribute to improve evidence-based decision making for the implementation of malaria eradication plans.

## THE STUDY

### Design, population and study área

A descriptive study was conducted in the Colombian Caribbean region. This region is located in northern Colombia and is made up of the departments of Atlántico, Bolívar, Cesar, La Guajira, Magdalena, Sucre and the Archipelago of San Andrés, Providencia and Santa Catalina. It has an area of 107,027 km2 and represents 9.4% of the Colombian territory. The region includes 167 municipalities and has an estimated population of 8,900,000 inhabitants, 22% (1,963,548 inhabitants) of whom are at potential risk of malaria, and of these, 13% (260,544 inhabitants) are in areas with active transmission and presence of vectors.

### Study variables and inclusion criteria

We selected and included the universe of confirmed cases of uncomplicated malaria reported by the Departmental Health Secretariats to the Public Health Surveillance System (SIVIGILA) during the study period, as registered in the Integrated Social Protection Information System (SISPRO) (https://www.sispro.gov.co/Pages/Home.aspx). In addition, secondary information from the National Malaria Program for the period 1960-1999, available at the Ministry of Health and Social Protection was included.

We used the official case definition of uncomplicated malaria. The number of malaria cases and variables of place (departments) and time (year, decades and period) were used. In Colombia, it is mandatory that all malaria cases reported to the surveillance system be confirmed using parasitologicaldiagnosis by microscopy, rapid diagnostic tests or polymerase chain reaction. Microscopy examination is the gold standard for diagnosis of the disease in the country ^8^. Information on the population at risk was obtained from national census projections of the National Administrative Department of Statistics (DANE) (http://www.dane.gov.co/) for the years 1964, 1973, 1985, 1993, 2005 and 2018, corresponding to the rural population of the region. Malaria transmission intensity criteria by eco-epidemiological regions were assumed according to the classification established in a previous study ^6^. The Caribbean region, like the other regions, is made up of departments and municipalities that share similar social and environmental characteristics.

### Data analysis

The data of the variables were stored and analyzed with the Microsoft 365® Excel software. The distribution of quantitative variables was evaluated using the Kolmogorov-Smirnov

test. We constructed absolute frequency indicators such as total and specific cases. We calculated measures of central tendency such as mean number of cases and median according to the distribution and measures of relative frequency as general malariometric indices such as API (annual parasite index) and specific ones such as AFI (annual P. falciparum

index) and AVI (annual P. vivax index): [No. cases (total or

by parasite species x 1000) / (Population at risk) ]. We determined percentage distributions of cases per decade and per

department. Finally, dispersion measures such as standard deviation (SD), maximum and minimum values were used. The distribution by age group was estimated from the cumulative percentage distribution in the country during the 2010-2019 period. The coefficient of determination (R2) was

estimated in order to establish the time trend.

### Ethical Aspects

We followed the ethical requirements established in Resolution 8430 of 1993 (Article 11) of the Colombian Ministry of Health and Social Protection, which defines this research

as risk-free and therefore it does not require approval by an

ethics committee ^10^.

## FINDINGS

Between 1960 and 2019, 155,096 malaria cases were registered

in the Caribbean Region. The mean number of cases per decade was 25,849.3 SD: 4,192.3). The decades 1990-1999 (31,815 cases) and 1980-1989 (29,286 cases) had the highest number of cases. Among the departments, Bolivar with 43.4% (67,330 cases), La Guajira with 14.4% (22,330 cases) and Sucre with 12.1% (18,827 cases) contributed to the highest burden in the region (Table 1).

**Table 1:** Total malaria cases by decade in the Colombian Caribbean eco-epidemiological region, 1960-2019.

Departments	Decades						Total	Distribution % by department
	1960-1969	1970-1979	1980-1989	1990-1999	2000-2009	2010-2019		
Atlantico	457	1644	1012	10 200	419	169	13,901	9,0
Bolívar	6977	8571	12 215	15 352	4016	20 199	67,330	43,4
Cesar	2045	3049	1958	784	9251	241	17,328	11,2
La Guajira	819	3334	4544	3263	8539	1 831	22,330	14,4
Magdalena	7726	2747	1908	819	1936	226	15,362	9,9
Archipiélago de San Andrés, Providencia y Santa Catalina	0	0	0	1	0	17	18	0,01
Sucre	2030	5302	7649	1396	1616	834	18,827	12,1
Total	20 054	24 647	29 286	31 815	25 777	23 517	155,096	100
Distribución % por década	12,9	15,9	18,9	20,5	16,6	15,2	100	-

**Figure 1:**
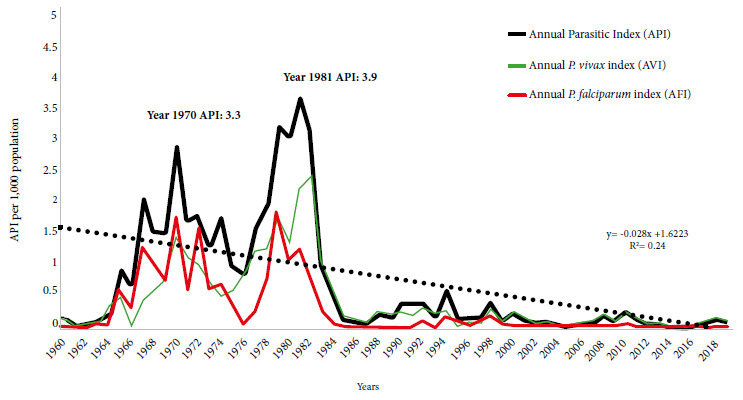
Malaria behavior in the eco-epidemiological region of the Colombian Caribbean, 1960-2019.

The secular behavior of transmission in the Caribbean Region showed a significant downward trend, with a variable endemic-epidemic, low-intensity pattern. *P. vivax* infections predominated. The most important outbreaks occurred in 1970 (API: 3.3 per 1,000 population), followed by 1981 (API: 3.9 per 1,000 population). The coefficient of determination (R2) was 0.24 (Figure 1).

**Figure 2:**
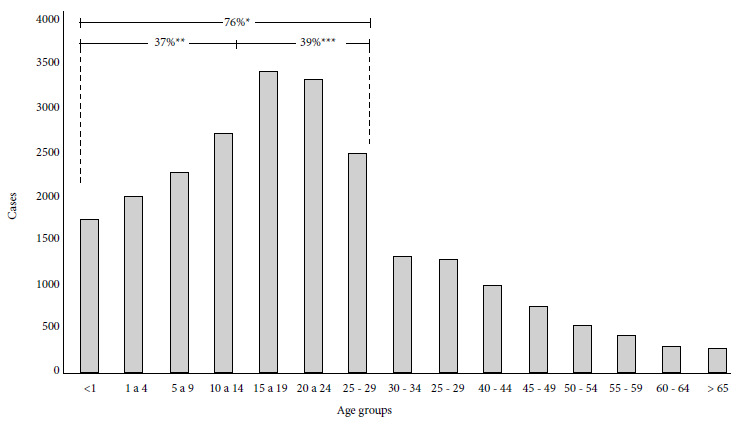
Distribution and percentage of malaria cases by age group, 2010-2019.

Sixty-two percent (96,072/155,096) of the registered malaria cases in the region were P. vivax, except in the departments of Cesar and Magdalena where *P. falciparum *infections predominated with 55.3% (9,574/17,328 cases) and 52.6% (8,086/15,362 cases), respectively (Table 2). Cases were found in all age groups, and the most vulnerable people were those under 29 years of age. These contributed 76% (17,802/23,517 cases) of the cumulative burden of cases registered in the 2010-2019 decade. Of the latter, the 15-29 age group was the most affected with 39% (9,148/23,517) of the cases (Figure 2).

**Table 2:** Distribution of cumulative malaria cases by parasite species in the departments of the Colombian Caribbean eco-epidemiological region, 1960-2019.

Department	*P. falciparum*		*P. vivax*		Total
	No. of cases	%	No. of cases	%	No. of cases
Atlantico	1366	9.8	12,535	90.2	13,901
Bolívar	23,520	34.9	43,810	65.1	67,330
Cesar	9574	55.3	7754	44.7	17,328
La Guajira	9503	42.6	12,827	57.4	22,330
Magdalena	8086	52.6	7276	47.4	15,362
Archipelago of San Andrés, Providencia y Santa Catalina	7	38.9	11	61.1	18
Sucre	6968	37.0	11,859	63.0	18,827
Total	59,024	38.0	96,072	62.0	155,096

## DISCUSSION

This study showed that malaria in the Caribbean region is of low transmission intensity, with a variable endemic-epidemic pattern, with a significant downward trend and a predominance of *P. vivax *cases.

The territory presents suitable conditions of receptivity and vulnerability for transmission, such as changes caused by deforestation, increased number illicit crops and illegal mining, which favor the reproduction of *Anopheles* vectors ^11^. The described situation is similar to that observed in transmission scenarios in neighboring endemic countries in the Caribbean basin.

In Nicaragua, a variable endemic-epidemic pattern was described during the 2000-2019 period, with an initial downward trend between 2000-2007, maintaining low transmission levels until 2014, and then again showing an upward trend until 2019 with predominance of *P. vivax i*nfections ^12^. In Costa Rica, there was a significant downward trend in morbidity, low transmission and predominance of *P. vivax. *In recent years, there has been a moderate increase in the number of cases, with nearly 50% of cases being imported ^13^. In Panama, malaria cases are mostly reported in the Darien region, where the transmission is endemic-epidemic, focalized and with low-intensity, with *P. vivax *being the most frequent species. Indigenous people are the most affected group, mainly those between 10 and 49 years of age. In addition, there is a permanent flow of immigrants from different countries and continents that increase vulnerability ^14^. In Venezuela, a re-emergence ofmalaria transmission has been reported in recent years, nonetheless, in the coastal states of Sucre and Zulia the intensity of transmission is low, with a prevalence of *P. vivax *in more than 90% of the cases, affecting the economically active population ^15^.

In Colombia, the eco-epidemiological regions of the Pacific, Urabá-Bajo Cauca Sinú San Jorge and Amazonia are the ones that contributed most to the malaria burden in the country between 2010-2019 ^6^. In the Caribbean region, active and focal transmission has only been described in municipalities in southern Bolivar and La Guajira, where occasional epidemic outbreaks are reported. In Bolívar, transmission is explained by the migration of susceptible populations and parasite carriers from other endemic areas in Colombia, due to social and political conflicts as well as economic interests, such as illegal gold mining^ (^ ^11^. The latter has been a fundamental factor in the transmission of malaria in that area, where the vectors An. darlingi, An. nuneztovari, and An. neomaculipalpus^ (^ ^16^ ^) ^have been reported.

The department of La Guajira, the northernmost department of Colombia, is characterized by its environmental, social and cultural complexity, which have influenced the dynamics of vector-borne diseases. The behavior of the malaria is of low intensity, with sporadic epidemic outbreaks, such as the one that occurred between December 1999 and February 2000. During those months, there was an unusual increase in the number of cases, most of which were caused by P. falciparum and affected the rural indigenous population. The main recognized breeding sites were bodies of water, ponds, “jaguayes” and swamps formed after the rainy season, where the immature forms of the *An.albimanus* and *An. triannulatus* vectors breed ^17^.

On the other hand, the predominance of *P. falciparum* in the departments of Cesar and Magdalena may have been related to the migration of people attracted by the cotton and banana agro-industrial growth between the 1940s and 1960s ^18^, these people came from neighboring regions such as Urabá - Bajo Cauca Sinú San Jorge and the Pacific, contributing to the predominance of malaria due to *P. falciparum. *Recently, the proliferation and exploitation of illicit crops adjacent to the Sierra Nevada de Santa Marta sector and the worsening of the armed conflict have caused population displacements that explain the intensification of transmission ^19^. Another relevant fact is that although the rest of the departments in the region have adequate receptivity conditions and competent Anopheles vector species are reported, there is no active transmission of the disease ^20^.

The main limitation of this study is the low perception of the magnitude and importance of this problem in the region by those responsible of the situation, which could affect the availability and reliability of the information. In addition, it is likely that the low knowledge of health professionals in the region has an impact on the diagnosis and notification of cases, which could lead to underreporting. On the other hand, it was difficult to analyze the age variable because it was not uniformly registered in the different information systems we used. It was only possible to obtain this variable from the national reports of the event in the last decade.

This study provided a baseline on the epidemiology of malaria in the Caribbean region.

In conclusion, malaria in the Caribbean region has had an endemic-epidemic behavior, with very low transmission intensity and with the presence of delimited foci of active transmission that contribute the greatest cumulative burden of cases in the region.

We recommend developing micro-stratification studies in municipalities with active transmission, considering the feasibility and viability of malaria elimination in order to implement plans, programs and projects in the region. This will strengthen local capacity regarding the technical-operational response for the implementation of regular preventive and timely control programs. It will also strengthen continuing medical education programs to improve knowledge and practice in order to ensure timely care of cases requiring diagnostic and treatment services. The development of comprehensive information systems is required for the consolidation of epidemiological intelligence and decision making; and to lead social and sectoral empowerment. Finally, basic and applied research lines should be established on clinical, epidemiological, entomological, environmental, social, prevention and control measures, among others.
