# Supplementary material for: Malaria in the eco-epidemiological region of the Colombian Caribbean, 1960-2019
Source: Rev Peru Med Exp Salud Publica. 2022 Dec 5;39(4):463–8. doi: 10.17843/rpmesp.2022.394.11359 (PMC11397599; doi:10.17843/rpmesp.2022.394.11359)
Supplement: Translation

# Translation: Malaria en la región eco-epidemiológica del Caribe Colombiano, 1960-2019

**Authors:** Luis Acuña-Cantillo, Mario J. Olivera, Julio Cesar Padilla-Rodríguez

**Affiliations:** 1 Instituto Nacional de Salud de Colombia, Bogotá, Colombia; 2 Red de Gestión de Conocimiento, Investigación e Innovación en Malaria, Instituto Nacional de Salud de Colombia, Bogotá, Colombia.

**Keywords:** Malaria, Epidemiología, *Plasmodium vivax*, *Plasmodium falciparum*, Caribe, Colombia

## Abstract

La malaria en Colombia tiene un comportamiento heterogéneo y variable, entre las regiones. Para establecer su comportamiento epidemiológico en la región del Caribe colombiano entre 1960 y 2019 se realizó un estudio observacional, descriptivo y retrospectivo a partir de registros del Ministerio de Salud y otras fuentes secundarias. Se definieron variables epidemiológicas y se elaboraron medidas de frecuencia y tendencia central. Se registraron 155 096 casos. Las décadas con mayor número de casos fueron 1990-1999 (20,5%) y 1980-1989 (18,9%). El promedio de casos por década fue de 25 849,3. Los índices parasitarios más elevados se registraron en 1970 (3,3 por 1000 habitantes) y 1981 (3,9 por 1000 habitantes). La especie *Plasmodium vivax* fue la más frecuente y la mayoría de la carga por grupos de edad se registró en menores de 29 años, entre 2010-2019. La malaria presentó un patrón endemo-epidémico de baja y muy baja intensidad de transmisión, con una tendencia al descenso.

## INTRODUCCIÓN

En Colombia la malaria produce el 54,7% de la carga acumulada de casos por enfermedades transmitidas por vectores ^1^. En el último lustro se observó un cambio en la prevalencia de la especie parasitaria en el país, predominando *Plasmodium falciparum* ^2^ ^,^ ^3^. 

Esta enfermedad persiste como problema de salud pública, imponiendo una alta carga económica y social ^4^ ^,^ ^5^. Su transmisión es endemo-epidémica, heterogénea y varía de mediana a baja intensidad en las regiones eco-epidemiológicas distribuidas en el territorio nacional, con diferentes condiciones de receptividad y vulnerabilidad. 

En la región Caribe, la enfermedad es focalizada y de muy baja transmisión ^6^. En el 2018, contribuyó con el 3% de la carga nacional de malaria, y la mayoría de los casos son importados de otras regiones con distintos grados de intensidad de transmisión ^7^. A pesar de esto, son pocos los estudios realizados sobre el tema en la región, los cuales han sido puntuales y con limitada divulgación, dificultando la toma de decisiones basadas en evidencia para la definición y ejecución de estrategias de eliminación ^8^.

La intensificación de las interacciones ambientales, económicas, sociales, políticas y culturales de la última década, podrían cambiar la dinámica de transmisión en la región Caribe en los próximos años, favoreciendo la reemergencia, mantenimiento e intensificación en los niveles endemo-epidémicos en la región ^9^. Por todo lo anterior, el objetivo de este estudio fue establecer el comportamiento epidemiológico de la malaria en la región del Caribe colombiano entre 1960 y 2019.

**MENSAJES CLAVE**

Motivación para realizar el estudio: la información disponible sobre la epidemiología de la malaria en la región del Caribe colombiano está incompleta, poco sistematizada y su divulgación es limitada. Esto ha generado un desconocimiento en su magnitud y una baja percepción de su importancia como problema de salud pública.

Principales hallazgos: el comportamiento de la malaria es endemo-epidémico, de baja a muy baja transmisión, focalizado y con aparición de brotes irregulares. Predominan las infecciones por Plasmodium vivax.

Implicancias: los resultados este estudio contribuyen a mejorar la toma de decisiones basadas en evidencia, para la ejecución de planes de eliminación de la malaria.

## EL ESTUDIO

### Diseño, población y área de estudio

Se realizó un estudio descriptivo en la región Caribe colombiana. Esta región está situada al norte de Colombia y está constituida por los departamentos de Atlántico, Bolívar, Cesar, La Guajira, Magdalena, Sucre y el Archipiélago de San Andrés, Providencia y Santa Catalina. Tiene una extensión de 107 027 km^2^ y representa el 9,4% del territorio colombiano, presenta 167 municipios, con una población estimada de 8 900 000 habitantes, el 22% (1 963 548 habitantes) de esta se encuentra en riesgo potencial de malaria, y de estos, el 13% (260 544 habitantes), se encuentran en áreas con transmisión activa y presencia de los vectores.

### Variables de estudio y criterios de inclusión

Se seleccionó e incluyó el universo de casos confirmados de malaria no complicada notificados, en el periodo de estudio, por las Secretarías Departamentales de Salud al Sistema de Vigilancia en Salud Pública (SIVIGILA), consignada en el Sistema Integrado de Información de la Protección Social (SISPRO) (https://www.sispro.gov.co/Pages/Home.aspx). Además, se incluyó información secundaria del Programa Nacional de Malaria del periodo 1960-1999, disponible en el Ministerio de Salud y Protección Social.

Se asumió la definición oficial de caso de malaria no complicada. Se utilizó el número de casos de malaria y variables de lugar (departamentos) y tiempo (año, décadas y periodo). En Colombia, es obligatorio que todos los casos de malaria notificados al sistema de vigilancia se confirmen mediante diagnóstico parasitológico por microscopía, pruebas de diagnóstico rápido o reacción en cadena de la polimerasa. El examen por microscopía es el estándar de oro para el diagnóstico de la enfermedad en el país ^8^. La información sobre la población en riesgo se obtuvo de proyecciones de los censos nacionales del Departamento Administrativo Nacional de Estadística (DANE) (http://www.dane.gov.co/) de los años 1964, 1973, 1985, 1993, 2005 y 2018, correspondientes a la población rural de la región. Se asumieron los criterios de intensidad de transmisión de la malaria por regiones eco-epidemiológicas acorde a la clasificación establecida en un estudio previo ^6^. La región Caribe, al igual que las otras regiones, está constituida por departamentos y municipios que comparten características sociales y ambientales.

### Análisis de datos

Los datos de las variables se almacenaron y analizaron con el programa Excel de Microsoft 365®. Se evaluó la distribución de variables cuantitativas mediante la prueba de Kolmogorov-Smirnov. Se construyeron indicadores de frecuencia absoluta como los casos totales y específicos. Se calcularon medidas de tendencia central como promedio de casos y mediana de acuerdo con la distribución y medidas de frecuencia relativa como los índices malariométricos general como el IPA (índice parasitario anual) y específicos como IFA (índice por *P. falciparum* anual) e IVA (índice por *P. vivax* anual): [N° casos (totales o por especie parasitaria x 1 000) / (Población en riesgo)]. Se establecieron distribuciones porcentuales de casos por décadas y por departamentos. Finalmente, se definieron medidas de dispersión como desviación estándar (DE), valores máximos y mínimos. La distribución por grupos etarios se estimó a partir de la distribución porcentual acumulada en el país durante el periodo 2010-2019. Se estimó el coeficiente de determinación (R^2^) para establecer la tendencia temporal. 

### Aspectos éticos

El estudio tuvo en cuenta los requisitos éticos establecidos en la Resolución 8430 de 1993 (Artículo 11) del Ministerio de Salud y Protección Social de Colombia, donde se define que son investigaciones sin riesgo y que no requieren aprobación de un comité de ética ^10^.

## HALLAZGOS

Entre 1960-2019, se registraron 155 096 casos de malaria en la Región Caribe. La media de casos por década fue de 25 849,3 DE: 4192,3). En las décadas de 1990-1999 (31 815 casos) y 1980-1989 (29 286 casos) se registraron los mayores números de casos. Con relación a los departamentos, Bolívar con 43,4% (67 330 casos), La Guajira con 14,4% (22 330 casos) y Sucre con 12,1% (18 827 casos) contribuyeron a la mayor carga en la región (Tabla 1).

**Tabla 1:** Casos totales de malaria por década en la región eco-epidemiológica del Caribe colombiano, 1960-2019.

Departamentos	Décadas						Total	Distribución % por departamento
	1960-1969	1970-1979	1980-1989	1990-1999	2000-2009	2010-2019		
Atlántico	457	1644	1012	10 200	419	169	13 901	9,0
Bolívar	6977	8571	12 215	15 352	4016	20 199	67 330	43,4
Cesar	2045	3049	1958	784	9251	241	17 328	11,2
La Guajira	819	3334	4544	3263	8539	1 831	22 330	14,4
Magdalena	7726	2747	1908	819	1936	226	15 362	9,9
Archipiélago de San Andrés, Providencia y Santa Catalina	0	0	0	1	0	17	18	0,01
Sucre	2030	5302	7649	1396	1616	834	18 827	12,1
Total	20 054	24 647	29 286	31 815	25 777	23 517	155 096	100
Distribución % por década	12,9	15,9	18,9	20,5	16,6	15,2	100	-

El comportamiento secular de la transmisión en la Región Caribe mostró una marcada tendencia al descenso, con un patrón endemo-epidémico variable de baja intensidad. Se observó un predominio de infecciones por *P. vivax*. Los brotes más importantes se registraron en 1970 (IPA: 3,3 por 1 000 habitantes), seguido por el de 1981 (IPA: 3,9 por 1 000 habitantes). Se registró un coeficiente de determinación (R^2^) de 0,24 (Figura 1).

**Figura 1:** Comportamiento de la malaria en la región eco-epidemiológica del Caribe colombiano, 1960-2019.

El 62% (96 072/155 096) de los casos de malaria registrados en la región fueron por *P. vivax*, excepto en Cesar y Magdalena donde predominaron las infecciones por *P. falciparum *con el 55,3% (9574/17 328 casos) y 52,6% (8086/15 362 casos), respectivamente (Tabla 2). Se registraron casos en todos los grupos de edad, siendo los grupos más vulnerables los menores de 29 años. Estos contribuyeron con el 76% (17 802/23 517 casos) de la carga acumulada de casos registrados en la década 2010-2019. Dentro de estos últimos, el grupo etario de 15-29 años fue el más afectado con el 39% (9148/23 517 casos) (Figura 2). 

**Tabla 2:** Distribución de casos acumulados de malaria por especie parasitaria en los departamentos de la región eco-epidemiológica del Caribe colombiano, 1960-2019.

Departamento	*P. falciparum*		*P. vivax*		Total
	N° casos	%	N° casos	%	N° casos
Atlántico	1366	9,8	12 535	90,2	13 901
Bolívar	23 520	34,9	43 810	65,1	67 330
Cesar	9574	55,3	7754	44,7	17 328
La Guajira	9503	42,6	12 827	57,4	22 330
Magdalena	8086	52,6	7276	47,4	15 362
Archipiélago de San Andrés, Providencia y Santa Catalina	7	38,9	11	61,1	18
Sucre	6968	37,0	11 859	63,0	18 827
Total	59 024	38,0	96 072	62,0	155 096

**Figura 2:** Distribución y porcentaje de casos de malaria por grupos de edad, 2010-2019.

## DISCUSIÓN

Este estudio demostró que la malaria en la región Caribe es de baja intensidad de transmisión, con un patrón endemo-epidémico variable, con marcada tendencia al descenso y con predominio de *P. vivax.*

El territorio presenta condiciones adecuadas de receptividad y vulnerabilidad para la transmisión, como cambios ocasionados por deforestación, la intensificación de los cultivos ilícitos y la minería ilegal, que favorecen la reproducción de vectores *Anopheles* ^11^. La situación descrita, es similar a la observada en escenarios de transmisión en países endémicos vecinos de la cuenca del Caribe. 

En Nicaragua, se observó en el periodo 2000-2019, un patrón endemo-epidémico variable, con una tendencia inicial al descenso entre los años 2000-2007, manteniéndose en bajos niveles de transmisión hasta el 2014, y nuevamente una tendencia al ascenso hasta el 2019 y predominio de las infecciones por *P. vivax* ^12^. En Costa Rica, se registró marcada tendencia a la reducción de la morbilidad, baja transmisión y predominio de *P. vivax*. En los últimos años se ha registrado un aumento moderado en la casuística, con cerca del 50% de casos importados ^13^. En Panamá, el principal foco de transmisión de malaria se encuentra en la región del Darién, donde existe transmisión endemo-epidémica, focalizada y de baja intensidad, prevaleciendo *P. vivax*. Los indígenas son los grupos más afectados, principalmente los de 10 a 49 años. Además, existe un flujo permanente de inmigrantes de distintos países y continentes que aumentan la vulnerabilidad ^14^. Mientras que en Venezuela en los últimos años se ha observado una reemergencia en la transmisión, pero en los estados costeros de Sucre y Zulia se ha mantenido una transmisión de baja intensidad, con una prevalencia de *P. vivax* mayor al 90%, y afectación de la población económicamente activa ^15^.

En Colombia, las regiones eco-epidemiológicas del Pacifico, Urabá-Bajo Cauca Sinú San Jorge y Amazonia son las que mas aportaron a la carga de malaria en el país entre 2010-2019 ^6^. En la región Caribe, solo se registra transmisión activa y focal en municipios del sur de Bolívar y La Guajira, donde se notifican brotes epidémicos ocasionales. En Bolívar, la transmisión se explica por la migración poblacional susceptible y portadores de parásitos procedentes de otras áreas endémicas en Colombia, debido a conflictos sociales, políticos e intereses económicos, como la explotación minera aurífera ilegal ^11^. Esta última, ha sido un factor fundamental en la transmisión de malaria en esa zona, donde los vectores reportados son *An. darlingi*, *An. nuneztovari*, y *An. neomaculipalpus* ^16^.

El departamento de La Guajira, el más septentrional de Colombia se caracteriza por su complejidad ambiental, social y cultural, las cuales han incidido en la dinámica de las enfermedades transmitidas por vectores. El comportamiento de la enfermedad de baja intensidad, con aparición de brotes epidémicos esporádicos, como el ocurrido entre diciembre de 1999 y febrero de 2000. Durante esos meses se presentó un aumento inusitado en el número de casos, la mayoría producidos por *P. falciparum* y afectó la población rural indígena. Los principales sitios de cría reconocidos fueron cuerpos de agua, charcas, jaguayes y pantanos formados después de la temporada de lluvias, donde se crían las formas inmaduras de los vectores *An. albimanus* y *An. triannulatus* ^17^.

Por otra parte, el predominio de *P. falciparum* en los departamentos de Cesar y Magdalena pudo haber tenido relación con la migración de personas atraídas por el desarrollo agroindustrial algodonero y bananero entre los años 40 y 60 ^18^ las cuales procedían de regiones vecinas como el Urabá - Bajo Cauca Sinú San Jorge y Pacífico, contribuyendo al predominio de la malaria por *P. falciparum*. Recientemente, la proliferación y explotación de cultivos ilícitos adyacentes al sector de la Sierra Nevada de Santa Marta, y la agudización del conflicto armado ocasionaron desplazamientos poblacionales que explicarían la intensificación de la transmisión ^19^. Otro hecho relevante es que a pesar de que en el resto de los departamentos de la región presentan condiciones adecuadas de receptividad y se reportan especies de vectores *Anopheles* competentes no existe transmisión activa de la enfermedad ^20^. 

La principal limitación de este estudio es la baja percepción de la magnitud e importancia de este evento en la región que tienen los responsables del tema, esto pudo incidir en la disponibilidad y confiabilidad de la información. Adicionalmente, es probable que el bajo conocimiento de los profesionales de la salud en la región incida en el diagnóstico y la notificación de los casos, lo que podrían ocasionar cierto subregistro. Por otra parte, se presentó dificultad para analizar la variable edad debido a que, su registro no era uniforme en los diferentes sistemas de información del periodo estudiado. Esta solo se pudo obtener de los informes nacionales del evento de la última década. 

Este estudio generó una línea de base sobre la epidemiologia de la malaria en la región Caribe.

En conclusión, la malaria en la región Caribe ha sido un evento endemo-epidémico, de muy baja intensidad de transmisión y presencia de focos delimitados de transmisión activa que aportan la mayor carga acumulada de casos en la región.

Se recomienda desarrollar estudios de microestratificación en municipios con transmisión activa; considerar factibilidad y viabilidad de eliminación de malaria e implementar planes, programas y proyectos en la región. Esto fortalecerá la capacidad local en la respuesta técnico-operativa para la implementación de programas preventivos regulares y de control oportuno. También fortalecerá los programas de educación médica continuada para mejorar el conocimiento y la práctica que garantice la atención oportuna de los casos que demanden servicios de diagnóstico y tratamiento. Se requiere el desarrollo de sistemas integrales de información para la consolidación de la inteligencia epidemiológica y la toma de decisiones; y liderar el empoderamiento social y sectorial. Finalmente, se deben establecer líneas de investigación básica y aplicada sobre los aspectos clínicos, epidemiológicos, entomológicos, ambientales, sociales, medidas de prevención y control, entre otras.
